# Medical News

**Published:** 1855-09

**Authors:** 


### MEDICAL NEWS.

Dr. Jacob Bigelow has resigned his post of Physician to the Massachusetts General Hospital. Dr. Augustus Gould was unanimously elected to fill the vacancy.

—

Dr. B. Fordyce Barker has been elected one of the Physicians to the Bellevue Hospital, New York, in the place of Dr. Thomas F. Cock, resigned.

—

Dr. J. C. O'Neal, of Baltimore, obtained a verdict from the Superior Court of that city for damages, in the sum of $10,000, against one of the persons who were injured by the awful railway accident of the 4th of July, last year. The defendant had both his legs fractured, and was treated by Dr. O'Neal. After his recovery, he circulated a report, through the papers, that his limbs had not been broken, and that Dr. O'Neal had used deception in the management of his case. Hence the verdict.— *Western Lancet.*

—

### OBITUARY.

Died, at Saratoga Springs, New York, on the 20th of August, Dr. Moreton Stillé, in the 33d year of his age.

It is with sorrow that we announce the above melancholy event to our readers. Dr. Stillé's acquirements, manly character, unassuming deportment, and moral worth secured for him the respect and esteem of all who were acquainted with him. With most excellent natural abilities, he had a carefully cultivated mind, was master of several languages, and from advantages of study and travel, was both an accomplished scholar and physician. As a writer, besides occasional contributions to the American Journal of Medical Sciences, and to this Journal, Dr. Stillé was author of a most valuable paper on the pathology of Cyanosis, written for his thesis for the degree of Doctor of Medicine, and honored by being published by authority of the Faculty of the University of Pennsylvania. It is unnecessary for us to comment on the merits of that paper, as it is well known to the profession, and secured for its author, both at home and abroad, a reputation rarely attained by one of his early age.

For the last two years of his life, Dr. Stillé had been unintermittingly occupied in writing a work on Medical Jurisprudence, which we are happy to state is printed, and will shortly be published. Early in the present year he was appointed Lecturer upon the Theory and Practice of

Medicine in the *Philadelphia Association for Medical Instruction.* A rare critical sagacity applied to the results of extensive research rendered his instructions both accurate and complete, and we are informed by his colleagues that they were highly esteemed by the class to whom they were imparted.

There is every reason to think that the confinement and sedentary life, resulting from his deep interest in and close application to his posthumous work, together with the new and onerous duties of his Lectureship, were of great injury to a constitution and frame physically unfitted for such untiring labor.

By his many friends, his memory will long be warmly cherished, and while we cannot cease to regret the bright promise blighted by his untimely death, we

> "Trust that those we call the dead
> Are breathers of an ampler day
> For ever nobler ends."

---

Died, at Richmond, on the 15th of July, of valvular disease of the heart, Dr. R. L. Bohannan, Professor of Obstetrics in the Medical College of Virginia, aged 68 years.

---

On the 12th of July, at Smithfield, Rhode Island, Dr. Elisha Bartlett, aged 51 years, author of a work on Medical Philosophy, also of a Treatise on Fevers, both of them highly valued by the profession.

---

M. Valleix.—This distinguished physician died of malignant sore throat, on the 12th of July last, after a short illness. His remains were accompanied to the cemetery of Mont Parnasse by a large number of devoted friends. The orations at the tomb were delivered by MM. Barth, Goupil, Latour, and his old master Louis, who was much affected. M. Valleix was a man of great erudition, and intimately acquainted with the contemporary medical literature of England. To strangers he was accessible and polite, and to not a few in this our northern metropolis, his memory is endeared by many acts of friendship. His chief works are—the *Guide du Médicin Praticien,* the *Traité des Névralgies,* and the *Clinique des Maladies des Enfants nouveau-nés.—Edin. Med. Jour.*

